# What Really Matters for Supervision Training Workshops? A Realist Evaluation

**DOI:** 10.1097/ACM.0000000000004686

**Published:** 2022-07-21

**Authors:** Van N.B. Nguyen, Charlotte E. Rees, Ella Ottrey, Corinne Davis, Kirsty Pope, Sarah Lee, Susan Waller, Claire Palermo

**Affiliations:** 1 **V.N.B. Nguyen** is a research fellow, Monash Nursing and Midwifery, Faculty of Medicine, Nursing and Health Sciences, Monash University, Clayton, VIC, Australia; ORCID: https://orcid.org/0000-0002-0982-2532.; 2 **C.E. Rees** is head of school, School of Health Sciences, College of Health, Medicine and Wellbeing, The University of Newcastle, Callaghan, NSW, Australia, and adjunct professor, Monash Centre for Scholarship in Health Education, Faculty of Medicine, Nursing and Health Sciences, Monash University, Clayton, VIC, Australia; ORCID: https://orcid.org/0000-0003-4828-1422.; 3 **E. Ottrey** is a research fellow, Monash Centre for Scholarship in Health Education, Faculty of Medicine, Nursing and Health Sciences, Monash University, Clayton, VIC, Australia; ORCID: https://orcid.org/0000-0002-2979-548X.; 4 **C. Davis** is a PhD candidate, Department of Nutrition, Dietetics and Food, School of Clinical Sciences at Monash Health, Monash University, Clayton, VIC, Australia; ORCID: https://orcid.org/0000-0002-6343-2260.; 5 **K. Pope** is a lecturer, Department of Occupational Therapy, Faculty of Medicine, Nursing and Health Sciences, Monash University, Frankston, VIC, Australia; ORCID: https://orcid.org/0000-0002-0010-4091.; 6 **S. Lee** is a PhD candidate, Monash Centre for Scholarship in Health Education, Faculty of Medicine, Nursing and Health Sciences, Monash University, Clayton, VIC, Australia; ORCID: https://orcid.org/0000-0002-2781-3082.; 7 **S. Waller** is an adjunct senior research fellow, School of Rural Health, Faculty of Medicine, Nursing and Health Sciences, Monash University, Bendigo, VIC, Australia, and assistant professor, Department of Medical Education, College of Medicine and Health Sciences, United Arab Emirates University, Al Ain, UAE; ORCID: https://orcid.org/0000-0002-6309-0360.; 8 **C. Palermo** is director, Monash Centre for Scholarship in Health Education, Faculty of Medicine, Nursing and Health Sciences, Monash University, Clayton, VIC, Australia, and associate dean (teaching and learning), Faculty of Medicine, Nursing and Health Sciences, Monash University, Clayton, VIC, Australia; ORCID: https://orcid.org/0000-0002-9423-5067.

## Abstract

**Purpose:**

Supervision training supports health care supervisors to perform their essential functions. Realist evaluations are increasingly popular for evaluating complex educational interventions, but no such evaluations exist appraising supervision workshops. Building on an earlier realist synthesis of supervision training, the authors evaluated whether supervision workshops work, for whom and under what circumstances, and why.

**Method:**

The authors conducted a 2-stage realist evaluation during 2018–2019 to refine and develop program theory. The intervention involved half-day, face-to-face supervision workshops as part of an Australian state-wide government-funded program for health care and human services supervisors. Data collection involved realist interviews with 10 workshop developers (stage 1) and 43 supervisors (stage 2). The authors employed team-based data analysis using realist logic to refine and develop program theory by identifying contexts, mechanisms, outcomes, and context-mechanism-outcome configurations.

**Results:**

Despite their brevity, the supervision workshops had many reported benefits for supervisors (e.g., improved satisfaction) through various perceived mechanisms pertaining to pedagogy (e.g., mixed pedagogies), workshops (e.g., optimal duration), and individuals (e.g., supervisor engagement). However, they also yielded negative reported outcomes (e.g., suboptimal knowledge gains) brought about by assorted perceived mechanisms related to pedagogy (e.g., suboptimal peer learning), workshops (e.g., content irrelevance), and individuals (e.g., suboptimal facilitator competence). Such mechanisms were thought to be triggered by diverse contexts including supervisors’ levels of experience, sector, and workplace supervision cultures.

**Conclusions:**

While the findings partly support the realist synthesis of supervision training and previous realist evaluations of faculty development, this realist evaluation extends this literature considerably. Health care educators should employ mixed pedagogies (e.g., didactic teaching, peer learning), relevant content, optimal workshop duration, and competent/engaging facilitators. Educators also need to tailor workshops according to supervisors’ contexts including the sectors and supervision cultures in which supervision is practiced, and supervisors’ levels of experience (e.g., experienced supervisors appreciated workshop brevity).

Supervision training for health care and human services professionals matters. Such training can help supervisors enact effectively the 3 essential functions of supervision: helping supervisees learn, supporting supervisees, and ensuring that supervisees perform to standards. ^1^ Education is a complex intervention, ^2^ so realist approaches have become increasingly popular in academic medicine research over recent years. ^3–6^ However, to our knowledge, few have explored interventions relating to clinical supervision training. Some studies have conducted realist syntheses or evaluations of workplace supervision, ^2,7,8^ but only one study has conducted a realist synthesis of clinical supervision training. ^9,10^ Although numerous realist evaluations have been published exploring health education interventions, including coaching skills training, ^11^ mentoring, ^12^ leadership development, ^13^ faculty development, ^14–17^ interprofessional learning, ^18^ knowledge exchange, ^19^ and preceptorship, ^20^ none have conducted realist evaluations of supervision training. Therefore, building on a previous realist synthesis of clinical supervision training, ^9,10^ we report on a realist evaluation of supervision training workshops to explore whether they work, for whom and under what circumstances, and why.

## Background

### Rationale for using realist evaluation

We selected realist methodology for this evaluation, ^21^ largely because this approach focuses on identifying context-triggering mechanisms, which in turn generate outcomes. ^22–25^ A glossary of realist terms we employ throughout this article is presented in Supplemental Digital Appendix 1, at http://links.lww.com/ACADMED/B254. ^21,26–35^ Realist evaluations are characterized by 5 concepts:

- Embeddedness: of human action in social processes or realities;
- Mechanisms: explanations of how things work;
- Contexts: the contextual conditions which may enable or hinder mechanisms;
- Regularities: the patterns of interactions between, for example, contexts and mechanisms; and
- Changes: or outcomes generated by context-triggering mechanisms. ^21^

More specifically, realist evaluations seek to illuminate why interventions work or not by building and testing realist program theory, and identifying semipredictable patterns, also known as demi-regularities (DRs) between contexts, mechanisms, and outcomes. These semipredictable patterns are understood in terms of context-mechanism-outcome configurations (CMOCs). ^21^ This methodology is therefore appropriate to guide the testing of a realist synthesis program theory for short-duration supervision training. ^9,10^

### Program theory from realist synthesis

A previous realist synthesis of the supervision training literature included 29 publications based on 28 studies; with 19 reports focusing on short-duration interventions, defined as one-off interventions or those conducted within 1 week. Pertinent to the current study focusing on one-off workshops, this synthesis identified 10 DRs lettered A–J associated with short-duration interventions. These interventions largely focused on learning outcomes related to supervision knowledge and skills; were delivered face-to-face; and employed mixed pedagogies including didactic, active, and experiential learning. ^9^

Nine DRs focused on supervisor outcomes (7 with positive and 2 with negative outcomes), whereas the tenth related to supervisee outcomes. Regarding positive supervisor outcomes, DR-A–DR-D related to improved satisfaction, confidence, engagement, knowledge, and practices through the mechanism of mixed pedagogies involving didactic teaching, as well as active and/or experiential learning. DR-E related to improved supervisors’ satisfaction, knowledge, and practices through the mechanism of social relationships. DR-F related to improved supervisory practices through the mechanism of improved supervisor knowledge, skills, and/or attitudes. DR-G identified supervisors’ improved supervision practices through the mechanism of increased supervisor confidence and/or self-efficacy. Finally, the 2 DRs with negative supervisor outcomes were no improvements in supervisory skills through supervisors’ lack of engagement or reinforcement with training (DR-H) and poor supervisor engagement in training through insufficient protected time (DR-I). DR-J related to positive supervisee outcomes, i.e., supervisee development and well-being, through the mechanism of structured training.

**Figure 1:**
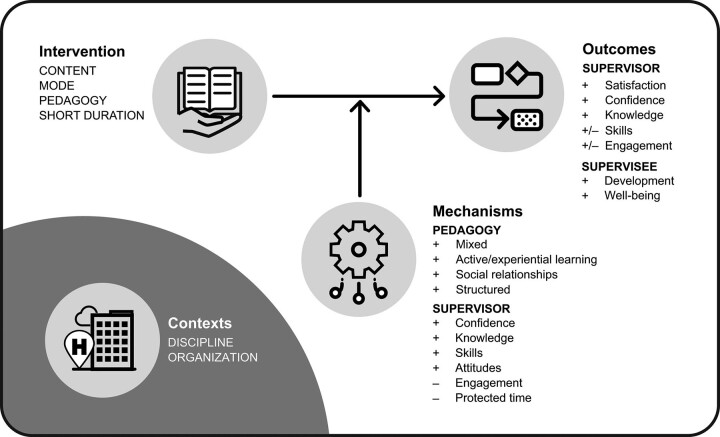
Visual representation of the realist synthesis program theory for how clinical supervision training works or not, reproduced from Rees et al ^9^ (https://creativecommons.org/licenses/by/4.0).

The program theory based on these 10 DRs for how short-duration supervision training interventions worked and why is depicted in Figure 1. However, this program theory lacked specificity in terms of the contexts thought to trigger these outcome-generating mechanisms. Indeed, disciplinary and organizational contexts were not found to be especially influential in this synthesis, probably because the studies had not been conducted using realist approaches, making it difficult to tease out contextual influences. ^9^ Therefore, the aim of this study’s realist evaluation is to develop this realist program theory by unpacking contexts, to better understand the extent to which a state-wide Australian supervision training program works, for whom and under what circumstances, and why.

## Method

### Intervention and setting

Funded by the Victoria State Government, we co-designed an evidence-informed, state-wide training program to enhance the supervision knowledge, confidence, and practice of 8,000 health care and human services workers within a funder-determined budget and time frame (2017–2021). Developed by experienced educators, this short-duration intervention—half-day face-to-face workshops—focused on introducing large numbers of supervisors across the state to introductory content; one workshop was directed at supervising students, and another focused on supervising colleagues. The workshops were designed to include mixed pedagogies involving didactic teaching, as well as active reflective and social learning. The workshops explored the diversity of supervision approaches across different disciplines and workplaces, drawing on Proctor’s model of supervision. ^1^ Both workshops covered the different functions of supervision, features of effective supervision, educational principles and practices to support effective supervision, and evaluation of supervisory practices. Delivered by facilitators from varying backgrounds in health care education and supervision, each workshop was limited to 40 participants. Supervisors across the state could register to attend 1 or both of the workshops at their preferred location, free of charge.

### Evaluation design

We applied a 2-stage evaluation approach to test the realist synthesis program theory. ^9^ The aim of stage 1 was to elucidate program developers’ thoughts about how the program might work or not, for whom, and why. In stage 2, we sought to understand supervisors’ perceptions of how the workshops worked or not, under what circumstances, and why. This report follows the RAMESES II reporting standards for realist evaluations. ^35^

### Sampling and recruitment methods

In both stages, we used maximum variation sampling to capture diverse perspectives, experiences, and supervisory contexts. ^36,37^ In stage 1, we invited staff from the funder and university who were involved in the design, development, management, and delivery of the supervision workshops to take part in a realist interview. We employed a combination of email and in-person contacts to approach potential participants. In stage 2, we employed email, video, and face-to-face invitations to recruit supervisors attending workshops at different locations. We explicitly encouraged supervisors from diverse professional backgrounds, with different levels of experience, and from varied workplace settings with different supervision cultures to participate. Eligibility criteria were supervisors who attended the workshop(s) and were currently or soon to be supervising students and/or colleagues.

### Data collection methods

We asked all participants to complete a personal details questionnaire recording demographic and professional characteristics. In both stages, the interviewers (S.L., V.N.B.N., C.D.) used realist interviewing techniques to elicit understandings about contexts, mechanisms, and outcomes in relation to the workshops. These techniques involved the interviewers seeking information, as well as presenting theories to aid their refinement or development while maintaining a natural flow of conversation. ^38–40^ The interviewers received feedback from 2 other authors (C.E.R., S.W.) on their interviewing skills to ensure consistency and adherence to the theory-driven nature of realist methodology. Audio-recorded interviews were conducted face-to-face, by telephone, or via Zoom according to participant convenience.

In stage 1, a single interviewer (S.L.) interviewed each program developer between August 2018 and December 2018. We asked participants about their expectations of the workshop outcomes, who they thought the workshop would work for or not and why, and the different outcomes that might occur in varying contexts. We conducted 10 interviews, which ranged from 48 to 109 minutes (average 68 minutes, total of 11 hours and 27 minutes). These interviews provided early information to refine and develop the realist synthesis program theory. ^9^ In stage 2, the other interviewers (V.N.B.N., C.D.) collected data from the workshop participants following their workshop attendance between May 2019 and September 2019. We aimed to elicit supervisors’ understandings and experiences of the workshops to further refine and develop the realist synthesis program theory. ^9^ Interview questions with supervisors focused on the contexts in which they undertook their supervision, their reported workshop outcomes, and why they thought the workshops contributed to these outcomes (mechanisms). We conducted 43 interviews (average 50 minutes, range 32–97 minutes), culminating in a total of 35 hours and 47 minutes of data. Note that we also collected longitudinal audio diaries and exit interviews from selected longitudinal cases in 2019 and 2020 to evaluate a different, extended-duration intervention; these data are presented elsewhere. ^41^

### Data analysis

All interviews were transcribed verbatim. We employed a team-based approach using realist logic, ^21^ to analyze data drawing on the 5 stages used in framework analysis. ^42^ Throughout these stages, we aimed to refine and develop program theories by identifying contexts, mechanisms, outcomes, and CMOCs.

#### Familiarization.

While the identification of CMOCs began during data collection, the formal analytic process began post data collection with the whole team familiarizing themselves with 2 diverse subsets of data by independently listening to audio-recordings and reading transcripts: 4 interviews from stage 1 and 8 interviews from stage 2. These subsets were diverse in that they included participants with different involvement in program development/implementation (stage 1) and with varied professional backgrounds, levels of experience, workplace settings, and supervision cultures (stage 2).

#### Developing the coding framework.

We developed a comprehensive coding framework partly based on the realist synthesis program theory, ^9^ as well as our initial analysis of the subsets of data. The resulting framework added new CMOCs to those already identified from the realist synthesis. ^9^ A copy of the coding framework is available from the corresponding author (C.E.R.) on request.

#### Coding data.

Two authors (V.N.B.N., E.O.) imported all transcripts into NVivo software, version 12 for Windows (QSR International, Melbourne, Australia), and coded contexts, mechanisms, outcomes, and CMOCs line-by-line as per the coding framework. We noted variations to the CMOCs using memos to assist decision making about refining and developing program theory. ^43^ We then compared our independent coding of 2 transcripts at the start of the coding process and subsequently verified the coding of 12 other transcripts to ensure coding consistency. Several of us (C.E.R., C.P., V.N.B.N., E.O., K.P.) met monthly to discuss any issues that arose during coding.

#### Charting data to establish patterns.

We used NVivo’s queries function to seek similarities, differences, and patterns in the outcomes and contributing context-triggering mechanisms. We synthesized the coded CMOCs to develop DRs, presenting these alongside causal statements and illustrative quotes. The whole team was included to provide multiple perspectives on the interrogation and theorizing of data.

#### Interpretation of data.

To interpret the data, we compared it with the realist synthesis program theory, ^9^ to refine and further develop realist program theory.

## Results

### Participant characteristics

In stage 1, all 10 program developers invited to participate consented. The majority identified as female (n = 8), aged 45–64 years (n = 6), and Oceanian (n = 6), defined as Australian and New Zealand Peoples by the Australian Bureau of Statistics. ^44^ Half possessed teaching qualifications (n = 5), the majority had a PhD (n = 6), a health professions education background (n = 6), moderate to expert supervisory expertise (n = 7), and moderate to expert program development expertise (n = 8). Two participants represented themselves as funders, 7 as workshop facilitators, 5 as program evaluators, and 2 as program managers. Note that some had multiple roles.

In stage 2, 51 supervisors expressed interest but only 43 eligible participants consented. These were recruited from 28 workshops in metropolitan (22 workshops) or regional areas (6 workshops). The majority identified as female (n = 34), aged 25–44 years (n = 27), and Oceanian (n = 25). They were mostly from the health care sector (n = 36), and the majority identified as experienced supervisors (n = 28) and described their workplace cultures as supervision-enabled (n = 24), i.e., workplaces with explicit supervision frameworks/arrangements, and organizational supports for supervisors and supervisees (non-enabled supervision cultures were considered as those without explicit supervision frameworks or arrangements or without managerial support for supervision). Ten attended the workshop about supervising students only, 18 attended the workshop about supervising colleagues only, and 15 attended both.

### Refining and developing realist program theory

**Figure 2:**
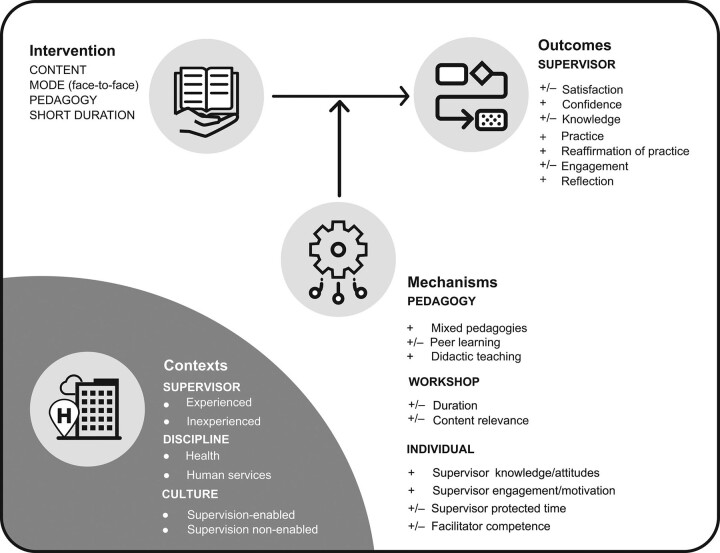
Visual representation of the modified program theory for how supervision workshops work or not (*Note:* the valence of mechanisms and outcomes are indicated as + (positive), − (negative), or +/− (both). Practice broadly encompasses supervision skills and behaviors).

In our testing of the 10 DRs identified in the realist synthesis program theory, ^9^ we refined 8 of them into 6 DRs, while finding no evidence for the remaining 2. However, we developed new realist program theory consisting of 6 additional DRs. Given the similarities across the data for stages 1 and 2, as well as our desire to present findings as parsimoniously as possible, we next present a synthesis of the 12 DRs based on the thrust of the mechanisms. Note that these 12 DRs (so-called semipredictable patterns; see glossary in Supplemental Digital Appendix 1, at http://links.lww.com/ACADMED/B254) are a synthesis of 76 CMOCs in our coding framework. As suggested by De Weger and colleagues, ^25^ we aim to present these rich and complex results as clearly and concisely as possible, and in a way most relevant to, and manageable for, academic medicine professionals. Interested readers can request a copy of our coding framework, including the 76 CMOCs, from the corresponding author (C.E.R.). Importantly, we found evidence of most contexts triggering outcome-generating mechanisms (i.e., workplace settings, supervision experience levels, workplace supervision cultures), so below we highlight only the contexts that appeared most likely to trigger outcome-generating mechanisms. These synthesized DRs, with illustrative quotes with contexts [C], mechanisms [M], outcomes [O], and valence [+, −, or +/−] identified, are supplied in Tables 1–3. Following the flexible approaches employed in realist studies, we present these CMOCs as causal statements in Tables 1–3 in the order of IOMC [intervention-outcome-mechanism-context] as we feel this is the most easily understandable sequence: “that this outcome was caused by this mechanism, which was in turn ‘triggered’ by this context.” ^25(p6)^ To further facilitate understanding, we visualize these configurations through our modified program theory, which can be seen in Figure 2.

### Pedagogy mechanisms (DR-1 through DR-4)

Four of the 8 DRs that we refined from the realist synthesis related to the mechanism of mixed pedagogies. ^9^ Firstly, we synthesized these 4 DRs into 1 refined DR based on our realist evaluation (see DR-1, Table 1). While the mechanism and positive workshop outcomes remained the same (i.e., satisfaction, confidence, engagement, knowledge, practice), we refined the context to extend beyond health care. We also found that this outcome-generating mechanism was most likely triggered within non-enabled supervision cultures (those without established supervision frameworks or organizational supports for supervisors). We also refined the realist synthesis DR with the mechanism of social relationships into 2 DRs with the mechanism of peer learning for our realist evaluation (see DR-2 and DR-3, Table 1). For DR-2, we found that the workshops yielded mixed outcomes (i.e., satisfaction, knowledge, reaffirmation of practice) through the mechanisms of optimal/suboptimal peer learning triggered by various contexts (see DR-2, Table 1). Regarding DR-3, we found the workshops yielded positive outcomes (i.e., confidence, practice, reflection) through positive peer learning in the health care sector (see DR-3, Table 1). Finally, we developed a new DR-4, which identified the positive workshop outcome of knowledge through the mechanism of didactic teaching (see DR-4, Table 1). This mechanism was mostly triggered within the health care sector in supervision-enabled cultures.

**Table 1:**
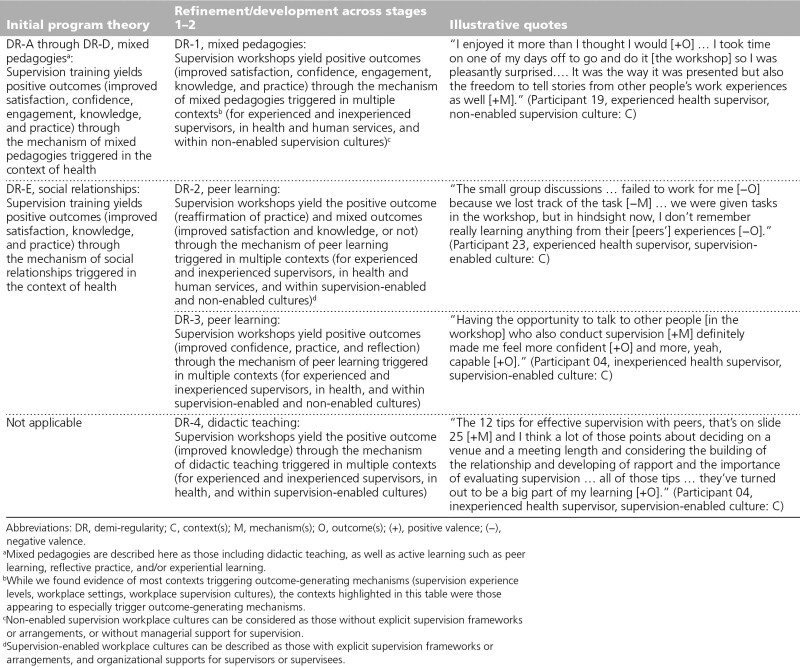
Refinement and Development of Realist Program Theory Involving Pedagogy Mechanisms From the Initial Program Theory Across Stages 1–2 of a Realist Evaluation, Including Illustrative Quotes, Victoria, Australia, 2018–2019

### Workshop mechanisms (DR-5 through DR-8)

Two new DRs were identified relating to the mechanism of workshop duration triggering either negative or positive outcomes (see DR-5 and DR-6, respectively, Table 2). Regarding DR-5, the workshops yielded insufficient knowledge gains through suboptimal workshop duration (i.e., too short) in the human services sector (see DR-5, Table 2). Concerning DR-6, the workshops yielded increased satisfaction through optimal workshop duration for experienced supervisors within non-enabled supervision cultures (see DR-6, Table 2). An additional 2 new DRs were identified relating to the mechanism of workshop content relevance, but with either negative or mixed outcomes (see DR-7 and DR-8, respectively, Table 2). Regarding DR7, the workshops yielded insufficient knowledge gains through suboptimal workshop content relevance to supervisor workplace context for inexperienced supervisors in the human services (see DR-7, Table 2). Concerning DR-8, the workshops yielded mixed outcomes in terms of knowledge gains through the mechanism of workshop content relevance to supervisor experience triggered by various contexts (see DR-8, Table 2).

**Table 2:**
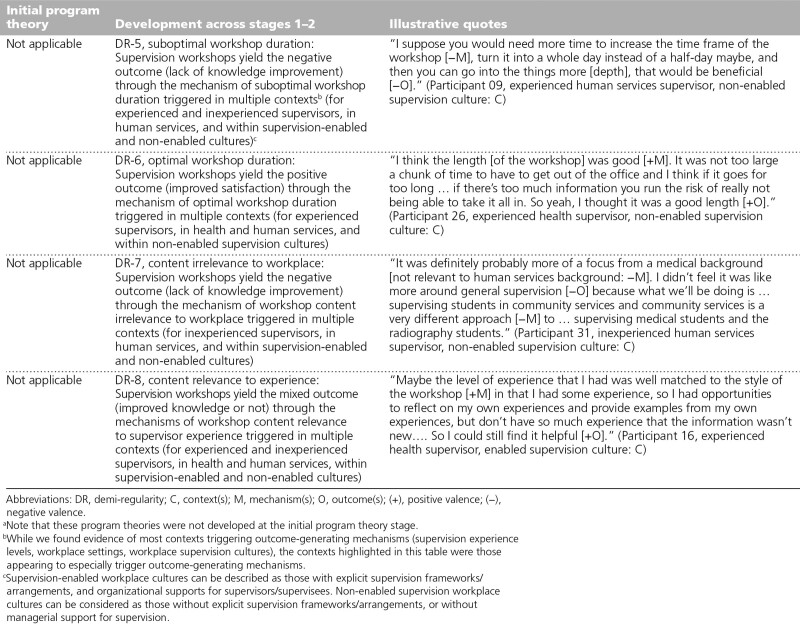
Development of Realist Program Theory Involving Workshop Mechanisms Across Stages 1–2 of a Realist Evaluation, Including Illustrative Quotes, Victoria, Australia, 2018–2019^a^

### Individual mechanisms (DR-9 through DR-12)

We refined 3 DRs from our realist synthesis pertaining to supervisor mechanisms. ^9^ Concerning DR-9, we refined the mechanism of supervisor knowledge, skills, and/or attitudes to just supervisor knowledge or attitudes, while the positive outcome remained the same (i.e., practice). However, we extended the original health care context to include the human services sector, and the mechanism also seemed most likely triggered in non-enabled supervision cultures (see DR-9, Table 3). Regarding DR-10, we switched the valence of both the mechanism (i.e., supervisor engagement or motivation) and outcomes, as well as extending those positive outcomes beyond practice, including satisfaction, knowledge, and engagement. We also extended the contexts beyond health care to include various contexts (see DR-10, Table 3). Concerning DR-11, the mechanism supervisor protected time and outcome engagement remained the same, although the valence shifted from negative to mixed, and we extended the context beyond health care to include various contexts (see DR-11, Table 3). Finally, we identified a new DR pertaining to the mechanism of facilitator competence. The workshops were thought to yield mixed outcomes (i.e., satisfaction, engagement, knowledge) through facilitator competence/incompetence for experienced supervisors (see DR-12, Table 3).

**Table 3:**
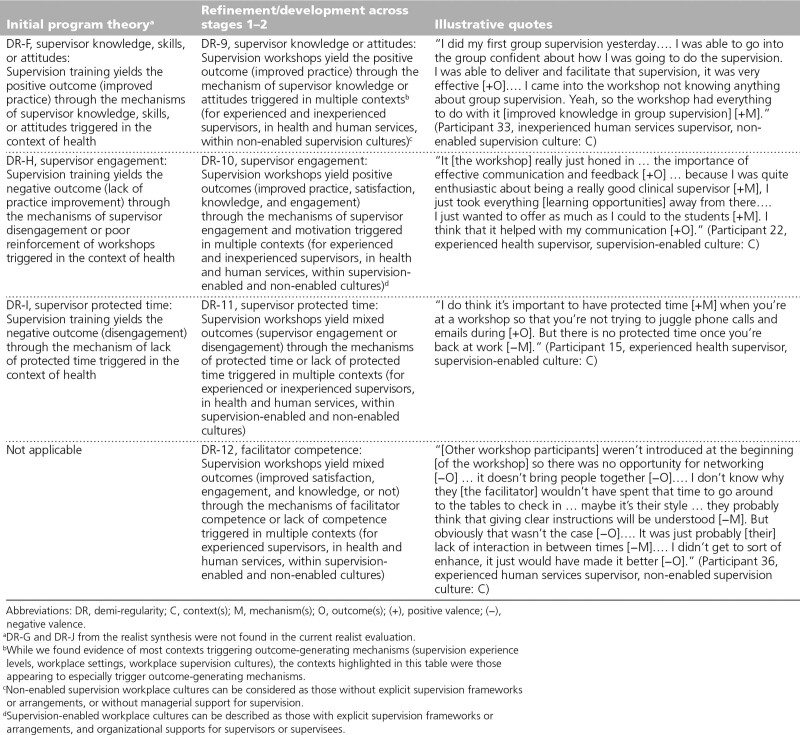
Refinement and Development of Realist Program Theory Involving Individual Mechanisms From the Initial Program Theory Across Stages 1–2 of the Realist Evaluation, Including Illustrative Quotes, Victoria, Australia, 2018–2019

## Discussion

While the workshops aimed to improve supervisors’ knowledge, confidence, and practice, our realist evaluation identified additional positive supervisor outcomes including satisfaction, engagement, reflection, and reaffirmation of practice. These positive outcomes were thought to be generated by mechanisms relating to pedagogy (i.e., mixed pedagogies, peer learning, didactic teaching), workshops (i.e., optimal duration, content relevance to supervisor experience), and individuals (i.e., supervisor knowledge or attitudes, engagement, and protected time, and facilitator competence). Workshop attendees also reported experiencing negative outcomes pertaining to satisfaction, knowledge, and engagement. These negative outcomes were thought to be triggered by mechanisms also related to pedagogy (i.e., suboptimal peer learning), workshops (i.e., suboptimal duration, content irrelevance to supervisor workplace/experience), and individuals (i.e., lack of protected time for supervisors, suboptimal facilitator competence). While these mechanisms were often triggered in assorted contexts including the health care or human services sectors, experienced or inexperienced supervisors, and supervision-enabled or non-enabled cultures, we found some context-specific program theories. Although these findings are partly comparable with the realist synthesis, ^9^ as well as other realist evaluations of short-duration faculty development interventions, ^15–17^ we discuss differences between our findings and previous literature here.

Regarding program outcomes, we found no evidence for supervisee outcomes, probably because we did not explore supervisees’ perceptions as other studies have done. ^45,46^ Furthermore, we did not identify positive supervisor outcomes (e.g., career progression) found in other faculty development realist evaluations, ^17^ possibly because our workshop duration was brief. However, we did identify novel positive supervisor outcomes (e.g., reflection, reaffirmation of practice), thereby extending previous realist synthesis findings. ^9^ Additionally, we identified novel negative workshop outcomes, extending previous faculty development realist evaluations that focused exclusively on positive outcomes only. ^15–17^

Concerning mechanisms, there were 2 mechanisms identified in the previous realist synthesis for which we found no evidence of (i.e., structured training, supervisor confidence/efficacy). ^9^ Structured training may not have been found as a mechanism in our evaluation because this was previously found as a mechanism for improved supervisee development and well-being, ^45,46^ and we did not identify supervisee outcomes in our evaluation. Furthermore, we did not find confidence/efficacy as a mechanism, probably because this mechanism was found previously for improved supervisory practices for a longer 40-hour intervention. ^47^ Moreover, our study did not identify positive mechanisms previously identified in faculty development realist evaluations such as professionalization, ^17^ again probably because our workshops were brief. However, we found a wider variety of mechanisms in our evaluation than previously identified, ^9^ including those relating to pedagogy (i.e., peer learning, didactic teaching), workshop (i.e., duration, content relevance), and individuals (i.e., facilitator competence). Although some of these have been found in previous realist evaluations of faculty development (e.g., interventions employing participatory approaches including reflective practice, having content relevant to faculty needs), ^11,15–17^ these were previously identified as contexts, not mechanisms. Furthermore, we also identified novel negative mechanisms (e.g., suboptimal peer learning, content irrelevance, suboptimal facilitator competence), extending previous faculty development realist evaluations focusing exclusively on positive mechanisms. ^15–17^

Finally, this evaluation extends the realist synthesis considerably by teasing out contexts that trigger outcome-generating mechanisms. That 2 mechanisms, optimal workshop duration and facilitator competence, were most likely triggered for experienced supervisors suggests that these supervisors may appreciate brief interventions and be more discerning about facilitator competence. ^16^ That 1 mechanism, content relevance to supervisor workplace, was most likely triggered for inexperienced supervisors suggests that workplace-related content relevance is especially important for those supervisors. ^13^ That 2 mechanisms, peer learning and didactic teaching, were most likely triggered in the health care sector, and 2, suboptimal workshop duration and content irrelevance to workplace, in human services, probably reflects differences in supervision cultures, training, and practices across different sectors. ^11,48^ For example, in health care contexts, individuals may prefer peer learning and didactic teaching. In human services contexts, individuals may prefer longer training targeted to their sector (the workshops were mostly underpinned by health literature). That several mechanisms (i.e., mixed pedagogies, optimal workshop duration, supervisor knowledge and attitudes) were most likely triggered in non-enabled supervision cultures highlights the value this brief workshop adds for supervisors with supervisory knowledge or attitudes, who experienced organizational backdrops without established supervision frameworks or managerial support for supervision. Finally, that a single mechanism, didactic teaching, was mostly triggered in supervision-enabled cultures suggests that supervisors with established supervision frameworks or managerial support derive more value from didactic teaching (i.e., the knowledge components of the supervision training). ^12,14,49^

### Limitations

Our study is not without its limitations; and these must be considered when thinking through the implications of our findings. For example, the contexts, mechanisms, outcomes, and CMOCs we identified are based on supervisors’ self-reports only, so we cannot be sure whether they concur with objective outcome measures such as supervisor knowledge tests, or supervisees’ perceptions of supervisor outcomes. Furthermore, supervisors reported outcomes for themselves, rather than their supervisees, so we still have limited understanding about the impact of workshops on supervisees. While we employed maximum variation sampling, most participants identified as female and Oceanian and came from the health care sector. Our findings are therefore less transferable to supervisors not identifying as female and those who are culturally and linguistically diverse, as well as those from human services sectors. Finally, although our findings are relatively concordant with the realist synthesis and other realist evaluations of short-duration faculty development programs, they are specific to our half-day supervision workshops designed for health and human services workers.

### Conclusions

Despite their brevity, supervision workshops have numerous reported benefits for supervisors through various mechanisms pertaining to pedagogy, workshops, and individuals, as well as within various contexts. Speaking to the mechanisms we identified, medical educators should consider employing several mixed pedagogies including peer learning and didactic teaching, content relevant to supervisors’ workplaces and experience levels, and optimal workshop duration commensurate with experience (e.g., experienced supervisors appreciated brief half-day workshops), and competent and engaging facilitators. However, educators do need to be mindful of contexts triggering those mechanisms, thereby tailoring workshops according to supervisors’ levels of experience, as well as the sectors and supervision cultures in which supervisors supervise. Again, regarding identified mechanisms, for academic medicine supervisors participating in training, we encourage several approaches: be motivated and engaged to embrace the opportunities afforded by workshops, capitalize on their existing knowledge and attitudes within workshops, and protect their time for workshop learning. Finally, for workplace employers, consistent with identified mechanisms, we encourage them to protect academic medicine supervisor time to enable them to engage maximally in supervision workshops. Further research is needed, employing mixed methods, to further refine/develop realist program theory. We encourage realist evaluators to include objective methods such as knowledge tests and subjective approaches with multiple stakeholders such as supervisors and supervisees in future research. We also encourage realist evaluators to include groups other than those that made up our participants, such as males and academic medicine professionals. Finally, this evaluation has focused on supervision training workshops only, and we know from the realist synthesis that intervention duration can lead to different outcomes triggered by different mechanisms within different contexts. Therefore, further realist evaluation is needed to explore extended-duration supervision training and to compare training of different durations. ^41,50^

## Acknowledgments:

The authors would like to acknowledge colleagues involved in the broader evaluation of the supervision training program: Vicki Edouard, Eve Huang, Charlotte Denniston, Keith Sutton, and Bernadette Ward. They thank all supervision training developers and supervisors who participated in this study for sharing their views and experiences.

## Supplementary Material


