# Supplementary material for: What Really Matters for Supervision Training Workshops? A Realist Evaluation
Source: Acad Med. 2022 Jul 21;97(8):1203–12. doi: 10.1097/ACM.0000000000004686 (PMC9311464; doi:10.1097/ACM.0000000000004686)
Supplement: Supplementary file 1 [file acm-97-1203-s001.pdf]

Supplemental Digital Appendix 1

Glossary of Realist Terms

Realist methodology: Underpinned by realist philosophy, realist research employs a generative understanding of causality, i.e., "the outcomes we observe are generated by causal processes and forces that we cannot see… and which operate (or not) according to contexts in which they occur."[27, p.1] Realist methodology has been described as a theory-driven approach that aims to explain the effectiveness of a program/intervention/policy by unpacking the relationships between contexts, mechanisms, and outcomes.[26]

Context: Context of a program/intervention/policy that includes multiple layers characterised by: individuals (stakeholders in the program/intervention/policy), interrelationships between individuals, institutional settings (rules, customs or norms related to the program/intervention/policy), and surrounding infrastructure (broader social, cultural or economic settings of the program/intervention/policy).[26]

Mechanism: Program mechanisms can be described as "an explanatory account of how and why programmes give rise to outcomes."[28, p.2] So, have been described as the underlying process, structure, entity, power or force that explains the relationship between contexts and outcomes of a program/intervention/policy.[29,30] Mechanism components are thought to include resources (defined as the component introduced in a context) and reasoning (described as participants' reasoning in response to the resource that alters their behaviour triggering outcomes).[31]

Change or *outcome:* A desired, observed or unexpected product of a program/intervention/policy.[32]

Context-mechanism-outcome configuration (CMOC): "a proposition stating what it is about a program which works for whom in what circumstances."[21, p.217]

Demi-regularity (DR): Can be defined as "semi-predictable patterns" or important and recurrent patterns of interaction between contexts, mechanisms and outcomes as found in the data.[33, p.13,34]

Program theory: A realist theory underpinning a program/intervention/policy about how and why it is expected to work in specific contexts.[35]

Copyright © the Association of American Medical Colleges. Unauthorized reproduction is prohibited.
